# Clinical Society of the New York Polyclinic Medical School and Hospital

**Published:** 1912-12

**Authors:** 


### CLINICAL SOCIETY OF THE NEW YORK POLYCLINIC MEDICAL SCHOOL AND HOSPITAL

(Meeting December 9th, 1912.)

DR. EARL CONNOR, JUVENILE CATARACT. (*Three Cases*).

Case 1.: 2,: 3. Three typical cases of juvenile Cataract in various traumatic and post-operatvie stages were shown in boys of 17, 11, and 5 years respectively. In the case where operation had been performed it was done for cosmetic purposes. The operation consists in producing a traumatic cataract and removing the lense. The results from the cosmetic point of view were satisfactory. Case 4. Ulcer of the cornea (malarial). A young Scotchman of 22 has a series of recurrent grayish opacities on his cornea which did not yield to the usual mode of treatment with the cautery. The ulcers were connected by a grayish line which involved the substantia propria corresponding to the sclerotic coat of the cornea. The patient had five or six relapses and treatment was very unsatisfactory until the cause was recognized. The local symptoms were those of pain, lacrymation and constitutional symptoms at times became prominent. A tentative diagnosis of malarial corneal ulcer was made and although no plasmodian was found in the blood, twenty or thiry grains of quinine per day together with Tr. of Ferri chloride were administered for six days at the end of which time ulcers and constitutional symptoms cleared up permanently. These cases are comparatively frequent in the tropics but very rare in this latitude and the case is of interest for this reason.

Case 5. *Acute Glaucoma.*—The patient a woman of 67 lost the sight of her left eye five years ago by glaucoma. Four days ago she began to complain of severe pain in her right eye and blurred vision. Redness was marked. Diagnosis of acute glaucoma was made and irridectomy performed. It is now three days after the operation and the patient who on admission could hardly tell the number of fingers held up before her eye is able to tell time. Dr. Conner urged the general practitioner to make an early diagnosis of acute glaucoma so that the patient's remaining vision could be saved.

Dr. John C. A. Gerster. Turn Buckle Lowman Clamp Method as a help in Lane Plating.

The doctor recalled that there were three possible types of fractures of the long bones: Transverse, oblique and comminuted. The freeing and reducing of the fragments takes the longest time and is the point least written about. The clamps are indicated in operative cases with displacement. They are slipped around the shaft of the bone and manipulated until apposition is obtained while an assistant makes sufficient traction to overcome shortening. The Lane plate can then be slipped in underneath the clamps in any position. In a three-piece fracture two pieces are first clamped and brought into alignment and these two treated as one piece and then placed in apposition with the third. A screw, one turn of which lengthens it in a left and right directions, serves to keep the clamps at any desired distance apart, and also permits their adjustment in different planes. In an oblique fracture it is at times very difficult to make fragments stay in place. The clamps are applied and fragments spread to overcome the one-half inch shortening necessary. In some transverse fractures few difficulties are met with in reduction and the use of the clamps can be dispensed with.

DR. C. G. KERLEY RUPTURED ECTOPIC GESTATION.

Dr. Kerley exhibited a four months old foetus and a Uterus which he removed from a patient, who up to the time of the operation had menstruated regularly. The nourishment of this foetus was derived not from the tube but from new vessels formed from the omentum; this gave rise to the much discussed question whether or not it is possible to have an abdominal pregnancy. In the author's opinion this pregnancy commenced as a tubal one, and later by absoption became abdominal.

Dr. Sturmdorff said that two forms of ectopic gestation, the cornual and the omental were the most serious. No authentic case of an abdominal pregnancy from the very beginning of fertilization is on record. We have no criterion to establish the fact that from the very first moment the ovum is abdominally implanted. It is surprising that many more cases do not terminate in tubal or abdominal pregnancies, when we consider the vicissitudes the ovum is subject to, before reaching the uterus. The question of immediate or deferred operation in ruptured ectopic has been much discussed lately. The speaker is greatly in favor of immediate operation since the danger to the life of the mother is very grave.

DR. JUDSON A. QUIMY. EXHIBIRION OF GASTRO-INTESTINAL RADIOGRAPHS

A number of plates were shown illustrating intestinal stasis due to kinks in the Hepatic flexture of the colon. Usually the result of old adhesions. This leads to the pyloric spasm, displacement of other organs, varied gastric symptoms, constipation, neurasthenia, and a whole trail of others. Dr. Bassler without wishing to depreciate the work of Dr. Quimby, said that we depened entirely too much upon radiographs for diagnosis. Too many men are doing this work without being qualified. This is especially so in Sanotoria. The X-ray gives an idea for diagnosis, which the case does not merit. We should depend more on the clinical diagnosis. For his own part the speaker said he was inclined to interpret conservatively changes in the position of the colon. This organ, is normally more subject to changes in position than any other organ in the body. Also it is difficult to diagnose colonic adhesions from these plates.

Dr. F. Kennedy. A Case of Cerebellar Tumor

The patient 37 years old, had enjoyed perfect health up to 12 months ago. He then began to complain of intense nausea and early morning vomiting. Later began to have unsteady gait, with lurching toward right side. Two months ago unsteadiness in the right hand became noticeable, and also frequent attacks of diplopia. The headaches were only slight but the nausea became more intense.

Physical examination: Pupils are equal, and react normally to light and accomodation. There is a marked nystagmus of the cerebellar type, that is, on looking toward the right the movements of the eyeball are slow and swinging, when eyes are turned to the left the movements are slower. Slight weakness of the right side of the face is present. Complete deafness of the right ear and taste sense markedly diminished on the right side. The eighth nerve on the right side is wholly destroyed. The twenty-first is affected and the seventh is beginning to be affected. Hypotonia on the right side of the body with increased reflexes on the right side are present. The above symptoms all point to a tumor growth at the ponto-cerebellar angle where the nerves leave the skull. These growth are usually non-malignant fibroma growing from the sheath of the eighth nerve. Operation is advisable. Success depends upon the method of removal.—*"W"*

